# A Safe Haven Through Attachment: A Dyadic Perspective on the Association Between Cumulative Childhood Trauma and Relationship Satisfaction

**DOI:** 10.1177/08862605241270013

**Published:** 2024-08-13

**Authors:** Mathilde Baumann, Marie-Ève Daspe, Claude Bélanger, Natacha Godbout

**Affiliations:** 1 University of Quebec in Montreal, QC, Canada; 2 University of Montreal, QC, Canada

**Keywords:** dyadic adjustment, childhood maltreatment, romantic attachment, resilience, polyvictimization

## Abstract

Cumulative childhood trauma (CCT) increases the risk of experiencing interpersonal problems and relationship distress in adulthood. However, not all CCT survivors experience such difficulties, and little research has investigated protective factors against relationship dissatisfaction in CCT survivors and their partners. Romantic attachment might be one such factor that could reduce the harmful effects of a CCT history on relationship satisfaction for both survivors and their partners. Using a dyadic perspective, this study aimed to examine the association between CCT and relationship satisfaction and to test the moderating effect of attachment avoidance and anxiety on this association. A sample of 501 couples was recruited through a Canadian survey firm. Canadian couples who had provided their telephone number were randomly selected to complete the short form of the Dyadic Adjustment Scale, the Experiences in Close Relationships Scale, and the Childhood Cumulative Trauma Questionnaire. The actor–partner interdependence moderation model was used to guide the analyses. Results showed that individuals’ and partners’ higher CCT was correlated with both partners’ lower relationship satisfaction. The analyses revealed a moderating effect of lower attachment avoidance on the link between individuals’ CCT and their own relationship satisfaction. Specifically, individuals’ CCT was significantly and negatively associated with relationship satisfaction at high levels of attachment avoidance, but unrelated to relationship satisfaction at low levels of attachment avoidance. The final model explained 31.4% of the variance in relationship satisfaction. Overall, the findings support the relevance of couple interventions that focus on romantic attachment to improve relationship well-being in couples where one or both partners have experienced CCT.

## Introduction

Childhood interpersonal trauma, which includes a history of physical, psychological, or sexual abuse, physical or psychological neglect, witnessing interparental physical or psychological violence, or peer bullying before the age of 18 (Godbout et al., 2017a), represents an important social and public health issue (Afifi et al., 2016; World Health Organization, 2006, 2020). Experiencing different types of childhood interpersonal trauma, or cumulative childhood trauma (CCT), is common in trauma survivors (Briere & Scott, 2015; Hodges et al. 2013). Epidemiological studies have found that 30% to 50% of survivors report having experienced two or more types of interpersonal trauma during childhood (Finkelhor et al., 2011; MacDonald et al., 2016; Stoltenborgh et al., 2015). In the general population, individuals report having experienced, on average, 2.5 to 3 different types of interpersonal trauma in childhood (Briere & Scott, 2015; Godbout et al., 2020).

Studies have shown that CCT is related to severe and complex long-term repercussions on well-being (Briere et al., 2008, 2016; Godbout et al., 2020). Specifically, CCT has been associated with various physical and mental health issues in adulthood, such as depression, anxiety disorders, physical health difficulties, substance abuse, psychopathology, and relationship difficulties (for reviews, see: Afifi et al., 2016; Jaffee, 2017; Vaillancourt-Morel et al., 2024; Zamir, 2022). The foundations for lifelong health and well-being are rooted in early experiences, making CCT a critical public health concern. Multiple studies suggest that a significant proportion of mental health problems and relationship difficulties across the life course may be related to CCT (Fink & Galea, 2015; Kirkbride et al., 2024; Strathearn et al., 2020; Zamir, 2022). Given these findings, it is essential to examine CCT to gain a better understanding of its repercussions in adulthood and how this may affect survivors’ relational well-being.

### CCT and Relationship Satisfaction

Because CCT typically occurs in trusting relationships (Briere et al., 2008; Godbout et al., 2013), it is likely to hinder survivors’ relational well-being in adulthood. Studies indicate that CCT survivors tend to report wariness about developing intimate relationships and difficulty maintaining satisfying relationships (for reviews, see: Vaillancourt-Morel et al., 2024; Zamir, 2022). In adults engaged in a romantic relationship, CCT has been found to be related to increased relationship conflict, violence, and dissolution (Dugal et al., 2018, 2019; Godbout et al., 2013), as well as to higher levels of relationship distress in adulthood (Bigras et al., 2015; Godbout et al., 2009, 2020; Peterson et al., 2018). Relationship satisfaction is defined as the feeling of being in a fulfilling and healthy relationship with a stable partner (Sabourin et al., 2005; Spanier, 1976) and is a key determinant of well-being and longevity (for a review, see: Kiecolt-Glaser & Wilson, 2017). A large body of empirical evidence consistently shows negative associations between exposure to different types of childhood interpersonal trauma and lower relationship satisfaction in adulthood (Bigras et al., 2015; Godbout et al., 2009, 2013; Peterson et al., 2018). The few studies that have examined CCT exposure also report that relationship satisfaction decreases as CCT increases (e.g., Godbout et al., 2020). However, these studies have rarely examined both relationship partners (Vaillancourt-Morel et al., 2024).

### The Importance of Using a Dyadic Perspective When Studying Relationship Satisfaction

Few dyadic studies have confirmed mutual influences between CCT history and both partners’ relationship satisfaction. In a study conducted among heterosexual couples, Vaillancourt-Morel et al. (2019) have found that individuals’ CCT history was correlated with their own and with their partners’ lower relationship satisfaction. In another study (Ruhlmann et al., 2018) examining relationship satisfaction among heterosexual couples where one partner is a survivor, higher CCT in women predicted lower levels of relationship satisfaction in men; in couples in which both partners are survivors, men’s higher levels of CCT predicted lower levels of relationship satisfaction for themselves and for their partners. These findings support the need to examine CCT among both partners using a dyadic perspective, where the couple is considered a reciprocal system. Yet, while dyadic studies are essential to examine the link between CCT and survivors’ partners’ relationship satisfaction, they are relatively scarce (for a review, see: Vaillancourt-Morel et al., 2024). Moreover, although the link between CCT and relationship distress is increasingly studied, not all survivors report relationship difficulties in adulthood, which highlights the need to study protective factors against CCT’s potential effects (Godbout et al., 2013; Ruhlmann et al., 2018; Vaillancourt-Morel et al., 2024). One such protective factor worth exploring is romantic attachment, given its central role in relationships (Mikulincer et al., 2002).

### Attachment Theory

Attachment theory stipulates that, in adulthood, the romantic partner becomes the principal attachment figure in a relationship that shares characteristics of the parent–child attachment relationship (e.g., physical contact, feelings of security, support, presence; Hazan & Shaver, 1987). Individuals’ attachment styles which develop with their primary attachment figures (e.g., parental figures) shape the development of cognitive schemas of themselves and of others that guide their expectations, needs, behaviors, and understanding of relationships throughout their lives (Mikulincer & Shaver, 2016; Mikulincer et al., 2002).

Romantic attachment is typically conceptualized along two dimensions: attachment anxiety and attachment avoidance (Hazan & Shaver, 1987; Simpson & Rholes, 2017). The former refers to negative internalized self-representations (e.g., doubts about self-worth), which generate a heightened sensitivity to cues of rejection and fears of abandonment, while the latter refers to negative internalized representations of others (e.g., perception that others are not trustworthy), which translate into a need for self-reliance and discomfort with emotional closeness. Insecure attachment involves high levels of attachment anxiety and/or avoidance, whereas a secure attachment refers to low scores on both dimensions (Lafontaine et al., 2015; Simpson & Rholes, 2017).

### The Role of Romantic Attachment in Relationship Well-Being

While no study has examined the protective role of romantic attachment against CCT’s effects on relationship satisfaction, a recent review indicates that more secure attachment may protect against the deleterious effects of childhood interpersonal trauma on health in adulthood (Meng et al., 2018). In addition, some studies may be useful to understand how romantic attachment may modulate the association between CCT and relationship satisfaction. For instance, a recent dyadic study found that attachment anxiety moderates the association between perceived support and relationship satisfaction (Labonté et al., 2022). More precisely, the association between men’s higher perceived support and their own relationship satisfaction was stronger among those with low-to-moderate levels of attachment anxiety (Labonté et al., 2022). While this research did not study survivors of childhood trauma and did not assess the role of attachment avoidance, it provides insight into the protective effect of a more secure attachment on relationship satisfaction for both partners. Another research study conducted with women survivors, showed that attachment anxiety moderates the relationship between childhood interpersonal trauma and intimate partner violence victimization in adulthood (Smith & Stover, 2016). Specially, exposure to childhood trauma was positively associated with intimate partner violence victimization when attachment anxiety was high, while this association was nonsignificant when attachment anxiety was low, which suggests that a more secure attachment may protect survivors from the impacts of childhood trauma on relational well-being in adulthood (Smith & Stover, 2016). However, this study focused on the experience of female survivors and on attachment anxiety only. Also, the cumulative nature of childhood trauma antecedents was not documented. Finally, despite promising results regarding the moderating role of romantic attachment on couples’ relationship satisfaction (Labonté et al., 2022) and on the negative effects in adulthood of childhood trauma (Smith & Stover, 2016), gaps remain in the empirical research regarding its role in the association between CCT and relationship satisfaction, especially with a dyadic perspective.

More secure attachment is linked to comfort with intimacy and more acceptance and understanding of one’s emotions and those of others (Mikulincer & Shaver, 2016), which can improve relationship satisfaction through better emotional regulation and conflict resolution skills (Simpson & Rholes, 2017). Hence, the negative impact of CCT might be lessened in individuals who feel more secure within their relationships because they may be better equipped to meet their attachment needs as well as those of their partners (Godbout et al., 2009; Mikulincer & Shaver, 2016). This suggests that a more secure attachment (i.e., low levels of anxiety and/or avoidance) may protect CCT survivors from experiencing high levels of relationship distress. However, this hypothesis is yet to be tested empirically.

### Objectives and Hypotheses

This study aimed to examine the links between both partners’ CCT and relationship satisfaction and to test the moderating effect of romantic attachment on these associations. We hypothesized that (a) an individual’s CCT would be negatively associated with both their own relationship satisfaction (i.e., actor effects) and (b) their partner’s (i.e., partner effects), and that (c) romantic attachment would moderate these associations. More precisely, we expected that lower attachment anxiety and avoidance would attenuate the associations between CCT and relationship satisfaction. All possible interactions between a person’s CCT and their own attachment and their partner’s attachment were examined.

## Method

### Procedure and Participants

Participants were randomly selected by a survey firm from a list of telephone numbers (landline and cellular) from the province of Quebec, Canada. Participants who were eligible and interested in participating in the study were offered the opportunity to complete the questionnaire over the phone with trained and experienced interviewers. Participants were required to meet the following criteria: reside in the province of Quebec, be at least 18 years old, be proficient in French or English, and be in an ongoing couple relationship for at least 6 months. In order to reach 500 couples (i.e., both partners responded to the survey) out of a list of 6,652 potential participants who met the inclusion criteria and expressed interest in participating, we achieved this objective after the first 1,485 individuals were contacted and completed the survey. We stopped recruitment at this stage. The study was approved by the University of Quebec in Montreal’s institutional research ethics board.

Given the dyadic perspective used in this study, both partners were required. The final sample consisted of 1,002 participants (501 couples) aged 18 to 88 (*M* = 50.27; *SD* = 13.23). Most participants were Canadian (93.2%, *n* = 934) and primarily spoke French (94.7%, *n* = 949). Nearly two out of five participants (39.4%; *n* = 395) completed college or professional education degrees and 41.6% (*n* = 417) obtained a university degree. Most participants were currently employed (64.6%, *n* = 647), while 26% (*n* = 261) were retired. Personal annual income ranged from less than $20,000 CAD to over $100,000 CAD, with most participants (57%, *n* = 572) earning $60,000 CAD or less. All participants were identified as heterosexual, although recruitment was inclusive of sexual and gender diversity. Regarding relationship status, 57.9% of couples were married (*n* = 290), 39.1% (*n* = 196) were cohabiting, and 1.6% (*n* = 8) were not cohabiting. The average relationship length was 23 years (*SD* = 14; 0–72 years) and participants had an average of two children (*SD* = 1.39; 0–11).

### Measures

#### Cumulative Childhood Trauma

CCT was measured using the Childhood Cumulative Trauma Questionnaire (Godbout et al., 2017a), a self-report 17-item questionnaire assessing 8 types of childhood interpersonal trauma experienced before the age of 18: psychological, physical, and sexual abuse, physical and psychological neglect, witnessing psychological and physical interparental violence, and bullying by peers. For sexual abuse, participants indicated whether they had experienced unwanted sexual contact or sexual activity before 18 years of age with a person at least 5 years older or in a position of authority (three yes/no questions). Regarding the other eight types of trauma, participants were asked to report their respective frequency “in each typical year of childhood” using a seven-point Likert scale ranging from 0 (*never*) to 6 (*almost every day*). Based on the criminal code of Canada, participants were considered survivors of childhood interpersonal trauma if they reported at least one type of trauma before the age of 18. Each type of trauma was then dichotomized (0 = *not experienced* and 1 = *experienced in each typical year*). Items were summed to produce a total CCT score ranging from 0 (*no trauma*) to 8 (*all types of trauma*), with higher scores indicating higher cumulative exposure to multiple types of trauma. This measure’s internal consistency was high in previous studies (e.g., Bolduc et al., 2018; Godbout et al., 2020) as well as in the present study (α = .84 for women and .85 for men).

#### Relationship Satisfaction

Relationship satisfaction was assessed using the short form of the Dyadic Adjustment Scale (Sabourin et al., 2005; Spanier, 1976). Three items were scored on a six-point scale ranging from 0 (*never*) to 5 (*always*). The fourth item assesses the degree of relational happiness on a seven-point scale ranging from 0 (*extremely unhappy*) to 6 (*perfectly happy*). Scores obtained on all items were summed to obtain a total score ranging from 0 to 21, with higher scores reflecting higher levels of relationship satisfaction. A score of 13 represents the threshold used to differentiate dissatisfied individuals from those who are satisfied with their relationships (Sabourin et al., 2005). Internal consistency was acceptable in previous studies (α = .76–.96; Sabourin et al., 2005) and in the current sample (α = .71 for women and .68 for men).

#### Attachment

Romantic attachment was measured using the 12-items Experience in Close Relationships Scale (Lafontaine et al., 2015), which assesses attachment anxiety (6 items) and attachment avoidance (6 items). Responses were rated on a scale ranging from 1 (*disagree*) to 7 (*agree*). Mean scores were calculated for each subscale, with higher scores indicating higher levels of attachment anxiety or avoidance. Cut-offs have been suggested for significant levels on each subscale (above 3.5 for anxiety and above 2.5 for avoidance; Lafontaine et al., 2015). Internal consistency was acceptable in the validation study (α_anxiety_ = .87, α_avoidance_ = .84; Lafontaine et al., 2015) and in this study (α_anxiety_ = .84 for women and men; α_avoidance_ = .75 for women and .70 for men).

### Data Analysis

Data were screened for missing values, and there were no missing data in the sample of the present study. Descriptive and correlational analyses were performed using SPSS (v.27, IBM). To test hypotheses 1, 2 (i.e., associations between both partners’ CCT and relationship satisfaction), and 3 (i.e., moderating effects of attachment anxiety and avoidance on these associations), structural equation modeling based on the Actor-Partner Interdependence Moderation Model (APIMoM; Cook & Kenny, 2005; Garcia et al., 2015) was conducted using Mplus v.7 (Muthén & Muthén, 1998–2015). APIMoM allows us to account for the interdependence of partners’ data while simultaneously examining actor (i.e., the association between a person’s CCT and their own relationship satisfaction) and partner (i.e., the association between a person’s CCT and their partner’s relationship satisfaction) effects (Cook & Kenny, 2005; Garcia et al., 2015). All independent variables (i.e., CCT, attachment anxiety, and attachment avoidance) as well as all possible interactions were entered into the same model. Nonsignificant paths were removed in the final model to increase statistical power and parsimony. Age, relationship duration, cohabitation status (i.e., cohabiting/noncohabiting), and number of children were entered as potential covariates of relationship satisfaction. Nonsignificant variables were removed from the final model.

Before specifying the final model, an omnibus test of indistinguishability was conducted to determine whether partners were distinguishable by gender (Kenny et al., 2006). This test compares a freely estimated model to a model with equality constraints across women and men on means, variances, and intra- and interindividual covariances (Kenny et al., 2006). A chi-square difference test was performed using the rescaled 2 log likelihood difference test (Kenny et al., 2006). A significant omnibus test (*p* < .05) suggests that dyad members are distinguishable by gender (i.e., results differ between women and men), in which case parameters for women and men need to be freely estimated in the final model. By contrast, a nonsignificant omnibus test (*p* > .05) indicates that dyad members are indistinguishable by gender, which warrants the use of equality constraints in the model and the labeling of partners as partner 1 and partner 2. Model fit was examined using the Comparative Fit Index (CFI), the Tucker–Lewis Index (TLI), the Root-Mean-Square Error of Approximation (RMSEA), the Standardized Root Mean Square Residual (SRMR), and the chi-square index. A nonsignificant chi-square, CFI, and TLI greater than 0.90, and RMSEA and SRMR lower than 0.08 reflect an adequate model fit (Hu & Bentler, 1999; Kline, 2016). Analyses were conducted using the Maximum Likelihood Robust standard errors estimation method (Yuan & Bentler, 2000), which is robust to nonnormal distributions such as those of CCT.

## Results

### Descriptive and Preliminary Statistics

As presented in Table 1, the majority of participants reported experiencing at least two different types of trauma (67.1% of women; 63.7% of men). Women reported an average of 2.59 type of CCT (*SD* = 1.93), and men reported 2.44 (*SD* = 1.91). Analyses revealed that in most couples, both partners reported a history of interpersonal trauma (70.1%; *n* = 351), while only one partner reported such a history in 25.5% of couples (*n* = 128), and neither partner reported a history of interpersonal trauma in 4.4% of couples (*n* = 22).

**Table 1.** Prevalence of Childhood Interpersonal Trauma and Cumulative Childhood Trauma (*n* = 1,002).

	Women		Men		Total Sample	
Type of Trauma	*n*	%	*n*	%	*n*	%
Childhood interpersonal trauma						
Physical violence	125	25	152	30.3	277	27.6
Psychological violence	170	33.9	152	30.3	322	32.1
Physical neglect	51	10.2	85	17	136	13.6
Psychological neglect	255	70.9	326	65.1	681	68
Interparental psychological violence	202	40.3	179	35.7	381	38
Interparental physical violence	49	9.8	43	8.6	92	9.2
Bullying by peers	202	40.3	255	44.9	427	42.6
Sexual abuse	142	28.3	59	11.8	201	20.1
Cumulative childhood trauma						
None	79	15.8	93	18.6	172	17.2
One type	86	17.2	89	17.8	175	17.5
Two types	95	19	97	19.4	192	19.2
Three types	96	19.2	83	16.6	179	17.9
Four types or more	145	29	139	27.8	284	28.4

Based on the threshold for relationship satisfaction (i.e., 13), analyses revealed that 7.4% of women (*n* = 37) and 7.8% of men (*n* = 39) reported relationship dissatisfaction. Based on the cut-off for romantic attachment, 31.5% of women (*n* *=* 158) and 29.4% of men (*n* *=* 147) presented attachment anxiety, and 33.3% of women (*n* = 167) and 39.7% of men (*n* = 199) reported attachment avoidance. Table 2 presents correlations, means, and standard deviations for all measures separately for men and women. Results of paired sample *t*-tests indicated no significant gender differences for CCT, avoidant and anxiety attachment, and relationship satisfaction.

**Table 2.** Correlations, Means, and Standards Deviations Among Study Variables.

Variables	1	2	3	4	5	6	7	8
1. CCT women	1							
2. Relationship satisfaction women	−.17***	1						
3. Anxiety women	.18***	−.22***	1					
4. Avoidance women	.05	−.47***	.06	1				
5. CCT men	.12**	−.13**	.07	.04	1			
6. Relationship satisfaction men	−.10*	.58***	−.15***	−.23***	−.17***	1		
7. Anxiety men	.05	−.23***	.19***	.13**	.10*	−.19***	1	
8. Avoidance men	.05	−.31***	.17***	.24***	.06	−.52***	.17***	1
*M*	2.59	17.17	2.84	2.29	2.44	17.33	2.80	2.38
*SD*	1.93	2.93	1.38	1.29	1.91	2.83	1.35	1.20

*Note.* CCT = Cumulative Childhood Trauma.

* *p* ≤ .05. **p ≤ .01. ***p ≤ .001.

### Final Model

The omnibus test indicated that dyad members were indistinguishable regarding gender (χ^2^[30] = 27.29; *p* = .61). Partners were therefore identified as “partner 1” and “partner 2,” and the final model was conducted with equality constraints on parameters across partners.

**Figure 1.**
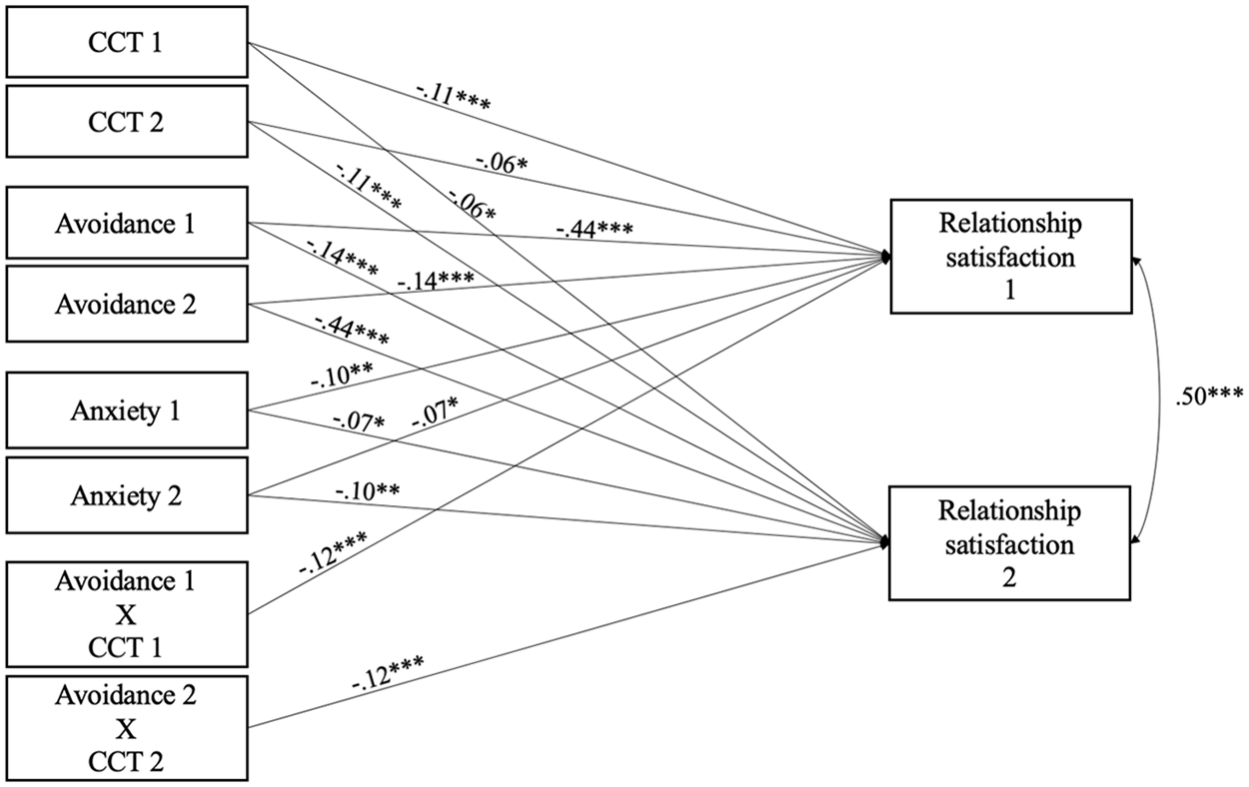
APIMoM final model of the moderating effect of attachment avoidance on the association between CCT and relationship satisfaction (*n* = 501). *Note*. Standardized coefficients are shown. All covariances between endogenous and exogenous variables were included in the final model, but not in the present figure. 1 = partner 1; 2 = partner 2; APIMoM = actor–partner interdependence moderation model; CCT = Cumulative Childhood Trauma. *p ≤ .05. **p ≤ .01. ***p ≤ .001.

A path analysis model examined the actor and partner associations between CCT and relationship satisfaction. Participants’ CCT (β = −.11; *p* ≤ .001) and partners’ CCT (β = −.06; *p* = .022) were found to be negatively linked to both participants’ relationship satisfaction. This model explained 4.1% of the variance in relationship satisfaction. All possible moderation effects were then tested (i.e., interaction effects of links between actor–actor, actor–partner, partner–partner, partner–actor) for both attachment anxiety and avoidance, for a total of eight interaction terms. As presented in Figure 1, individuals’ CCT, attachment anxiety, and attachment avoidance were negatively related to their own and their partners’ relationship satisfaction. One significant interaction was found: participants’ own attachment avoidance moderated the link between one’s CCT and one’s relationship satisfaction (actor-actor). All covariates were nonsignificant and therefore not included in the model.

**Figure 2.**
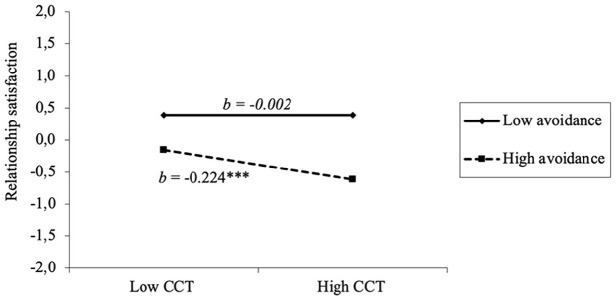
Moderation effect of attachment avoidance on the association between CCT and relationship satisfaction. *Note.* CCT = Cumulative Childhood Trauma. ***p ≤ .001.

More specifically, the test of simple slopes (see Figure 2) indicated that one’s own CCT was significantly and negatively associated with relationship satisfaction at high levels of avoidance (i.e., one standard deviation above the mean), but unrelated to relationship satisfaction at low levels of avoidance (one standard deviation below the mean). Attachment avoidance scores below 1.9 had a moderating effect on the link between CCT and lower relationship satisfaction. Results indicated excellent model fit (χ^2^[36] = 29.990, *p* = .75; CFI = 1.00; TLI = 1.007; RMSEA = 0.00, 90% CI [0.000, 0.023], SRMR = 0.04). The model explained 31.4% of the variance in relationship satisfaction.

## Discussion

This study aimed to explore the moderating effect of romantic attachment on the associations between CCT and relationship satisfaction from a dyadic perspective, in a random sample of couples from the general population. The results contribute to a growing body of research examining the long-term effects of childhood interpersonal trauma on relationship satisfaction in adulthood. Moreover, this study expands on existing research examining the negative impact of CCT on relationship satisfaction by using a dyadic perspective to examine the protective effect of romantic attachment.

### CCT and Relationship Satisfaction: Dyadic Effects

Results indicated that half of the participants experienced two or more types of interpersonal trauma in childhood, a prevalence rate similar to that found in meta-analyses (e.g., Stoltenborgh et al., 2015). In addition, in most couples (70.1%), both partners were CCT survivors, echoing previous studies on community samples (Dugal et al., 2019; Godbout et al., 2020). These findings attest to the magnitude of this problem and to a need to better understand its effects. Consistent with our hypotheses, we found that CCT was related to lower levels of relationship satisfaction for survivors and their partners. This is consistent with previous studies having investigated the deleterious effects of CCT on survivors’ relational sphere (for review: Vaillancourt-Morel et al., 2024). The proportion of variance explained by this direct relationship is relatively low, paralleling results from previous studies (e.g., Godbout et al., 2020). It is essential to note that interpersonal relationships are complex and influenced by a multitude of individual, relational, contextual, and cultural factors (Bühler et al., 2021; Hilpert et al., 2016; Karney & Bradbury, 1995; McNulty et al., 2021). Our results confirm that CCTs can have a significant direct impact on relationship satisfaction, thereby strengthening the current empirical knowledge.

### The Moderating Role of Attachment Avoidance

Attachment avoidance was found to moderate the link between higher CCT and lower relationship satisfaction. In essence, this link became nonsignificant among participants reporting low levels of attachment avoidance. This means that among survivors with low levels of avoidance, CCT did not have a negative impact on their relationship satisfaction. This result implies that having attachment avoidance below the cut-off (i.e., above 2.5; Lafontaine et al., 2015) is insufficient to buffer against the negative effects of attachment avoidance. Indeed, attachment avoidance needs to be quite low to buffer the effects of CCT on relationship satisfaction. The reduced manifestations of avoidant attachment (e.g., deactivation of the emotional system, increased need for self-reliance, discomfort with intimacy; Mikulincer & Shaver, 2016; Simpson & Rholes, 2017) at low levels of attachment avoidance may explain this moderating effect. In comparison to CCT survivors with high levels of avoidance, those with low levels of avoidance may feel more at ease disclosing their needs and emotions to their partners, which may enable them to offer and receive support more effectively (Godbout et al., 2017b). They may also be more comfortable with emotional closeness and be better equipped to acknowledge and understand their own emotions (Mikulincer & Shaver, 2016). These characteristics may shield them from—or even negate—the impacts of CCT on relationship satisfaction. Although previous studies have documented the role of low attachment anxiety in relational well-being (Labonté et al., 2022; Smith & Stover, 2016), this study contributes new insights by highlighting the protective role of a more secure attachment, namely low attachment avoidance, against the adverse effects of CCT in adulthood. No partner effect was observed regarding the moderating role of attachment avoidance. This could be attributed to survivors’ tendencies to have conflicting needs for both connection and isolation as they attempt to avoid distress and cope with trauma symptoms and related difficulties (e.g., distrusting others) (Ruhlmann et al., 2018; Simpson & Rholes, 2017). As such, avoidant attachment would influence the association between a person’s CCT and their own levels of relationship satisfaction. Contrary to our hypotheses, attachment anxiety did not moderate the link between CCT and relationship satisfaction. However, higher attachment anxiety was linked to lower levels of both one’s own and their partner’s relationship satisfaction, underscoring the influence of attachment anxiety on relational well-being (e.g., Godbout et al., 2017b). These results suggest that attachment anxiety could have a direct effect on relationship satisfaction, without directly interacting with a history of CCT. It is also possible that the stability which characterizes the couples in this study (i.e., older with an average age of 50.27 years and with an average relationship duration of 23 years) influences these results. Thus, the specific effects of attachment anxiety could be reduced in the context of a stable, long-term relationship, where, over time, attachment anxiety would not exacerbate the effects of CCT on their relationship. However, it is also plausible that the direct effect of attachment anxiety on relationship satisfaction remains significant and robust regardless of the trauma history, indicating that attachment anxiety might have a strong impact in various contexts.

Overall, this study enhances our understanding of why the association between CCT and relationship satisfaction is not consistently documented in the literature by providing new insights into the role of romantic attachment, an often overlooked moderating factor. The present findings can be interpreted through the theoretical lens of the vulnerability–stress–adaptation model (McNulty et al., 2021; Karney & Bradbury, 1995). This model conceptualizes the determinants of relationship stability according to three components: vulnerabilities (i.e., traits of each partner), stressors (i.e., external events, challenges faced by the partners), and adaptive processes (i.e., mechanisms for regulating relationship functioning and for coping with stressors and vulnerabilities). According to this model, CCT represents a vulnerability factor that can undermine relationship satisfaction. On the other hand, low attachment avoidance acts as an adaptive process as it provides a restorative basis to temper the impact of vulnerabilities (CCT) on survivors’ relationship satisfaction, thereby fostering resilience. Our findings point to the substantial positive influence that low attachment avoidance can have on relationship satisfaction, by offering a haven from which to adapt progressively in the aftermath of CCT.

### Limitations and Future Research

This study’s findings must be considered in light of its limitations. First, although anonymous self-report measures assessing traumatic experiences are considered reliable (Baldwin et al., 2019) and tend to be more accurate than other subjective measures (e.g., when participants need to self-identify as victims), they are susceptible to social desirability and recall bias, which could potentially influence the results (e.g., lower rates of trauma). Second, while the direction of associations was postulated based on temporal considerations (e.g., CCT occurring in childhood and relational satisfaction in adulthood) and theoretical grounds, it would be beneficial to confirm these findings using longitudinal data. Third, because we used a dyadic design, only couples where both partners agreed to participate were included in the study, which could introduce bias toward couples with more positive or stable relationships. This being said, it is important to highlight that the inclusion of a dyadic sample is a strength of this study, as it provides a more nuanced and comprehensive understanding of relational dynamics. However, the fact that the sample consisted mostly of older heterosexual couples, with an average relationship length of 23 years, implies that the findings may not be generalizable to other demographic groups (e.g., younger couples, sexually diverse couples, clinical samples, and different cultures and religions). The findings of this study should be replicated with more diverse samples and using longitudinal methods and mixed designs before being generalized to the broader population. In addition to the cumulative experience of different types of interpersonal trauma experienced in childhood, future studies may also focus on the characteristics and severity of each traumatic experience.

### Clinical Implications

This study carries clinical implications for professionals working with CCT survivors and couples experiencing relationship difficulties. The high prevalence of CCT and its documented effects on relationship satisfaction underscore the need for increased prevention efforts. This might involve educating professionals on the prevalence of CCT and its potential effects on intimate relationships, as well as on resources and trajectories toward recovery. Our findings emphasize the importance of training therapists and other professionals to consistently screen for various types of childhood interpersonal trauma in clients consulting for relationship difficulties. Moreover, professionals should offer trauma-sensitive interventions that consider both the individual and dyadic effects of these experiences on intimacy.

Our results also suggest that romantic attachment, particularly issues related to avoidant attachment, may be a key target for therapeutic treatment. Fostering a more secure attachment may promote higher relationship satisfaction in CCT survivors by attenuating CCT’s effect on relational well-being. Clinicians could introduce their clients to the concept of romantic attachment, as increased understanding of both partners’ attachment styles might enable them to better identify and respond to each other’s attachment needs, leading to increased relationship satisfaction. In the case of highly avoidant CCT survivors, a gradual exploration and acceptance of their emotions could be encouraged, with a focus on sharing emotions with their partners once they feel more secure in their relationships. This approach would allow partners to witness survivors’ vulnerability and better understand the attachment needs that underlie the avoidance behaviors that ultimately hinder the relationship’s well-being. As such, emotion-focused couple therapy (Greenberg & Johnson, 1988) tailored to trauma survivors (e.g., MacIntosh, 2019) may be a recommended approach.

## Conclusion

CCT can have lasting negative effects on adults’ intimate relationships, often resulting in interpersonal problems and reduced relationship satisfaction. Our study offers hope, showing that not all CCT survivors experience these adverse consequences. We found that higher levels of CCT in individuals and their partners were associated with lower relationship satisfaction. However, a protective factor emerged: lower attachment avoidance attenuated the negative impact of CCT on relationship satisfaction. Our results highlight the importance of developing interventions aimed at reducing attachment insecurities to promote the relational well-being of CCT survivors and their partners. This study reminds us that while the impact of childhood trauma is profound, the power of increased secure attachment (low attachment anxiety and avoidance) can illuminate a path toward healthier relationships, even for those who carry heavy burdens.
